# Bill of Mortality

**Published:** 1811-08

**Authors:** Lyman Spalding


*To the Editors of the Medical and Physical Journal.*

Gentlemen,

I HAVE in closed for your inspection the Portsmouth Bills of Mortality for 1809* and 1810. As you have done me the honour to publish those papers in your valuable Journal; and as they have become in this country the basis of calculations for annuities and insurance on lives, I think it proper to state to you that the Bill has now been published for ten years; and from the small number of inhabitants in this town, and my acquaintance with almost every family, I have been enabled to construct them with the greatest certainty of correctness.

The two last have been constructed with a view to determine what influence marriage has on longevity; for this purpose the married and unmarried have been characterized by specific marks.

I have yet to learn whether a Bill has been heretofore constructed on similar principles.

At some future period I intend to reprint the whole series, and as I have the materials by me, shall add to the former bills the characteristic marks of married and unmarried.

I have the honour to be, Gentlemen,

Yours, &c.

L. SPALDING.

*Portsmouth, Feb.* 10, 1811.

---

* The Bills received were each for 1810; being duplicates.
Editors.

BILL

### *BILL OF MORTALITY*

### For Portsmouth, Newhampshire, for A. D. 1810.

### BY LYMAN SPALDING, M. D.

| Complaint. | Age. | January. | February. | March. | April. | May. | June. | July. | August. | September. | October. | November. | December. | Total. |
|---|---|---|---|---|---|---|---|---|---|---|---|---|---|---|
| Apoplexy | 78‘ years | | 1 | | | | | | | | | | | 1 |
| Atrophy | 1, w 76‘ y 3. d 2, 5. 68‘ y 2, m 2 47‘ y 10. d 4. m 56, 2. y | 1 | 1 | 3 | | 2 | | 1 | | 3 | | 1 | 1 | 13 |
| Chlorosis | 13, years | | | | | | | | 1 | | | | | 1 |
| Cholera of infants | 4. m 2. 2, y 1, m 1. 1. 1, y | | | | | | | | 1 | 2 | 2 | 2 | | 7 |
| Consumption | 33. 55‘ 36. 23, 63‘ 35, 64‘ 58, 20, 21, 42‘ 37‘ 25‘ 52‘ 69‘ 40‘ 15, 41‘ 38‘ 41‘ 23. 28, 17, 25‘ 44‘ 28‘ 34. years | | 2 | 4 | 2 | 1 | 3 | 3 | 4 | 1 | 4 | 1 | 2 | 27 |
| Convulsions | 5, m 2, y 6. m 4. years | | | 2 | | | | | 1 | | | | 1 | 4 |
| Croup | 2, years | | | | | | | | | | | | 1 | 1 |
| Dropsy | 40‘ 60‘ 2. years | | | | | | 1 | | 1 | | | 1 | | 3 |
| Dropsy in the brain | 2. 13, y 5. m 2. 5, 4, 2, y 3, months | | 1 | 1 | 1 | 1 | | 1 | | 1 | 1 | | 1 | 8 |
| Erythema | 3. m 1, week | | 1 | | | | 1 | | | | | | | 2 |
| Fever inflammatory | 5. 4, years | | | | | | 1 | | 1 | | | | | 2 |
| Fever petechial | 9, years | | | | 1 | | | | | | | | | 1 |
| Fever puerperal | 18‘ 44‘ years | | | 1 | | | | | | | | | 1 | 2 |
| Fever pulmonic | 52‘ 75‘ 78‘ 5, y 4, months | 1 | 2 | | 1 | | | | 1 | | | | | 5 |
| Gout | 74· years | | | | | | | | | 1 | | | | 1 |
| Hemorrhage | 69· 52‘ years | | 1 | | | | | | 1 | | | | | 2 |
| Hooping cough | 1, 3, years | | | | | | | | | | 2 | | | 2 |
| Inflammation | 46· years | | | | | 1 | | | | | | | | 1 |
| Mortification | 53‘ 67‘ years | | | | | 1 | | 1 | | | | | | 2 |
| Old age | 89· 90‘ 77‘ 89‘ 90· 79‘ 78· years | 2 | 1 | | 2 | | | 2 | | | | | | 7 |
| Palsy | 58‘ 74· 87‘ 60‘ years | 1 | | 1 | | | | | | | 2 | | | 4 |
| Phrenitis | 50· years | | | 1 | | | | | | | | | | 1 |
| Quinsy | 7. m 1. 1, year | 2 | | | | | | | | 1 | | | | 3 |
| Scirrhus liver | 25. years | | | | | | | 1 | | | | | | 1 |
| Casualties: Burnt | 75‘ 4, 1, year | | | | | | | | | | 1 | 2 | | 3 |
| Casualties: Drowned | 2. 7. years | | | | | | | 1 | | | 1 | | | 2 |
| Casualties: Overlaid | 2. months | | | | | | | | | | | 1 | | 1 |
| Casualties: Run over by a waggon | 45· 5. years | | | | 1 | | | 1 | | | | | | 2 |
| Casualties: Suicide | 19. 25‘ years | | | | 1 | | | | | | | 1 | | 2 |
| BIRTHS {Males 130, Females 122} 252 | Still born 6 | | | | | | | | | | | | | |
| MARRIAGES 64. | Total | 7 | 10 | 13 | 9 | 6 | 6 | 11 | 11 | 9 | 13 | 9 | 7 | 111 |

Portsmouth, the Capital of the State of Newhampshire, situated 43° 5′ north latitude, and 6° 26′ east longitude from Washington, contains 6934 inhabitants.

The month of January exhibited the coldest, as well as the most remarkable change of weather ever recorded in Newhampshire. At 12 o'clock at noon of the 18th the thermometer was at 42°, and at 12 the 19th it had fallen 12° below zero. It fluctuated between 7° and 14° below zero from the 19th to the 22d. We do not find that either this sudden change, or extreme cold weather, had any material effect on the health of our citizens.

Note. This Bill is so constructed as to shew the complaint, age, sex, &c. and whether married or unmarried. When a period follows the age, it denotes the male sex, a comma the female sex; when in their usual place at the bottom of the line, unmarried—at the top of the line, married.

